# Increased Risk of Inflammatory Bowel Disease Among Patients With Ankylosing Spondylitis: A 13-Year Population-Based Cohort Study

**DOI:** 10.3389/fimmu.2020.578732

**Published:** 2020-10-02

**Authors:** Shuya Wang, Hsi-Kai Tsou, Jeng-Yuan Chiou, Yu-Hsun Wang, Zhiyi Zhang, James Cheng-Chung Wei

**Affiliations:** ^1^ Department of Rheumatology, The First Affiliated Hospital of Harbin Medical University, Harbin, China; ^2^ Functional Neurosurgery Division, Neurological Institute, Taichung Veterans General Hospital, Taichung, Taiwan; ^3^ Department of Rehabilitation, Jen-Teh Junior College of Medicine, Nursing and Management, Miaoli County, Taiwan; ^4^ School of Health Policy and Management, Chung Shan Medical University, Taichung, Taiwan; ^5^ Department of Medical Research, Chung Shan Medical University Hospital, Taichung, Taiwan; ^6^ Beijing Tsinghua Changgung Hospital, School of Clinical Medicine, Tsinghua University, Beijing, China; ^7^ Department of Rheumatology & Immunology, Chung Shan Medical University Hospital, Taichung, Taiwan; ^8^ Institute of Medicine, Chung Shan Medical University, Taichung, Taiwan; ^9^ Graduate Institute of Integrated Medicine, China Medical University, Taichung, Taiwan

**Keywords:** ankylosing spondylitis, inflammatory bowel disease, hazard ratio, musculoskeletal disorders, rheumatology, inflammatory

## Abstract

**Aim:**

Ankylosing spondylitis (AS) primarily affects the axial skeleton and extraarticular structures. Small-scaled studies have reported that the incidence and prevalence of inﬂammatory bowel disease (IBD) are higher in patients with AS than in the general population. This study determined the incidence of IBD in patients with AS using a large scale population-based cohort dataset.

**Methods:**

This was a retrospective cohort study. Patient data were collected from the Taiwan National Health Insurance Research Database from 2000 to 2012. We enrolled 3,804 patients with AS and 7,608 non-AS patients. The endpoint was IBD diagnosis by using International Classification of Diseases, Ninth Revision, Clinical Modification (ICD-9-CM) coding 555 and 556 after at least three outpatient visits or one hospital admission, until the end of 2012. The Kaplan–Meier *analysis was* performed to discriminate *the cumulative incidence of* IBD *and the log-rank test was used to test the significance. A* Cox proportional hazard model was used to estimate the hazard ratio (HR) for IBD between the AS and non-AS groups.

**Results:**

Among the population as a whole the Cox proportional hazard regression indicated that patients aged ≥65 years [adjusted HR (aHR): 2.48, 95% confidence interval (CI): 1.38–4.47] or with comorbidity of cancer (aHR: 3.51, 95% CI: 1.40–8.80) had a higher HR for IBD. Kaplan–Meier curves of cumulative incidence proportion of IBD indicated that patients with AS had a higher risk of IBD than the non-AS group in the subgroup aged <40 years (HR: 2.85, 95% CI: 1.51–5.40, p = 0.001).

**Conclusions:**

Patients with AS aged <40 years had a higher IBD risk than did those without AS in Taiwan. Clinicians and patients should be aware of this association.

## Highlights

Patients with AS aged <40 years had a higher IBD risk than patients without AS in Taiwan.

## Introduction

Inﬂammatory bowel disease (IBD), which includes Crohn’s disease (CD), ulcerative colitis (UC), and indeterminate colitis (IBD-unclassiﬁed), is a group of chronic relapsing inﬂammatory disorder affecting the gastrointestinal (GI) tract caused by a complex interaction of genetic factors, environmental exposures, and immune response dysregulation. CD can trigger inflammation in any part of the GI tract and results in various GI and systemic symptoms, such as abdominal pain, diarrhea, fistulae, weight loss, and anorexia. UC is characterized by inflammation in the colon, which leads to abdominal pain and subsequent diarrhea. However, IBD also displays certain extraintestinal manifestations. For instance, studies have reported an association between IBD and arthritis (1, 2).

Ankylosing spondylitis (AS) is a chronic inflammatory disease that affects the axial skeleton and extraarticular structures, such as eyes, skin, and bowels. IBD incidence and prevalence are higher in patients with psoriasis, psoriatic arthritis, and AS than in the general population.(3) The estimated overall incidence of IBD in patients with AS is 5%–10%, with approximately 60% of patients with AS presenting subclinical ileal inﬂammation (4). However, Wang et al. reported that the occurrence of IBD in Han Chinese patients with AS was rare.(5) Therefore, we examined the IBD risk in Taiwanese patients with AS using the large Taiwan National Health Insurance Research Database (NHIRD) and compared the risk in patients with AS with that in matched comparison cohort patients without AS.

## Materials and Methods

### Data Source

In this population-based retrospective cohort study, data were obtained from the NHIRD which enrolls approximately 99% of the 23 million beneficiaries in Taiwan. This database includes all the registrations and claims data, including diagnoses, drug prescriptions, inpatient care, outpatient visits, emergency hospitalization, and diagnoses according to the International Classification of Diseases, Ninth Revision, Clinical Modification (ICD-9-CM). Data collected from 2000 to 2012 were extracted for 1 million subjects, sampled from the 23 million beneficiaries. The 1 million longitudinal health insurance database was generated by randomly allocation to represent 23 millions whole population, provide by the Authority of National Health Insurance. The sampled database was de-identified as the Longitudinal Health Insurance Database (LHID), and the study was approved by the Institutional Review Board of Chung Shan Medical University Hospital.

### Study Group and Outcome Measurement

Patients diagnosed as having AS between January 1, 2000, and December 31, 2012, were selected from the LHID (n = 4007). Patients receiving their first diagnosis of AS (ICD-9-CM code 720.0) after at least three outpatient visits or one hospitalization, were considered to be newly diagnosed as having AS. The date of the first AS diagnosis was set as the index date. Patients diagnosed as having inflammatory bowel disease (ICD-9-CM 555 and 556) before the index date were excluded (n = 203). The non-AS group comprised 988084 individuals who were never diagnosed as having AS, randomly selected from 1999 to 2013. A ratio of 1:8 was used to match the 30,432 comparison cohort to patients with AS based on age and sex. The index date for the non-AS group was set as the index date of their AS group counterpart.

Follow-up was begun on the index date and ended on the IBD diagnosis date, December 31, 2012, or the date of withdrawal from the national insurance system. New cases of IBD were identified from the database by the presence of records of ICD-9-CM code 555 or 556 with at least three outpatient visits or one hospitalization.

### Covariates and Matching

**Figure 1:**
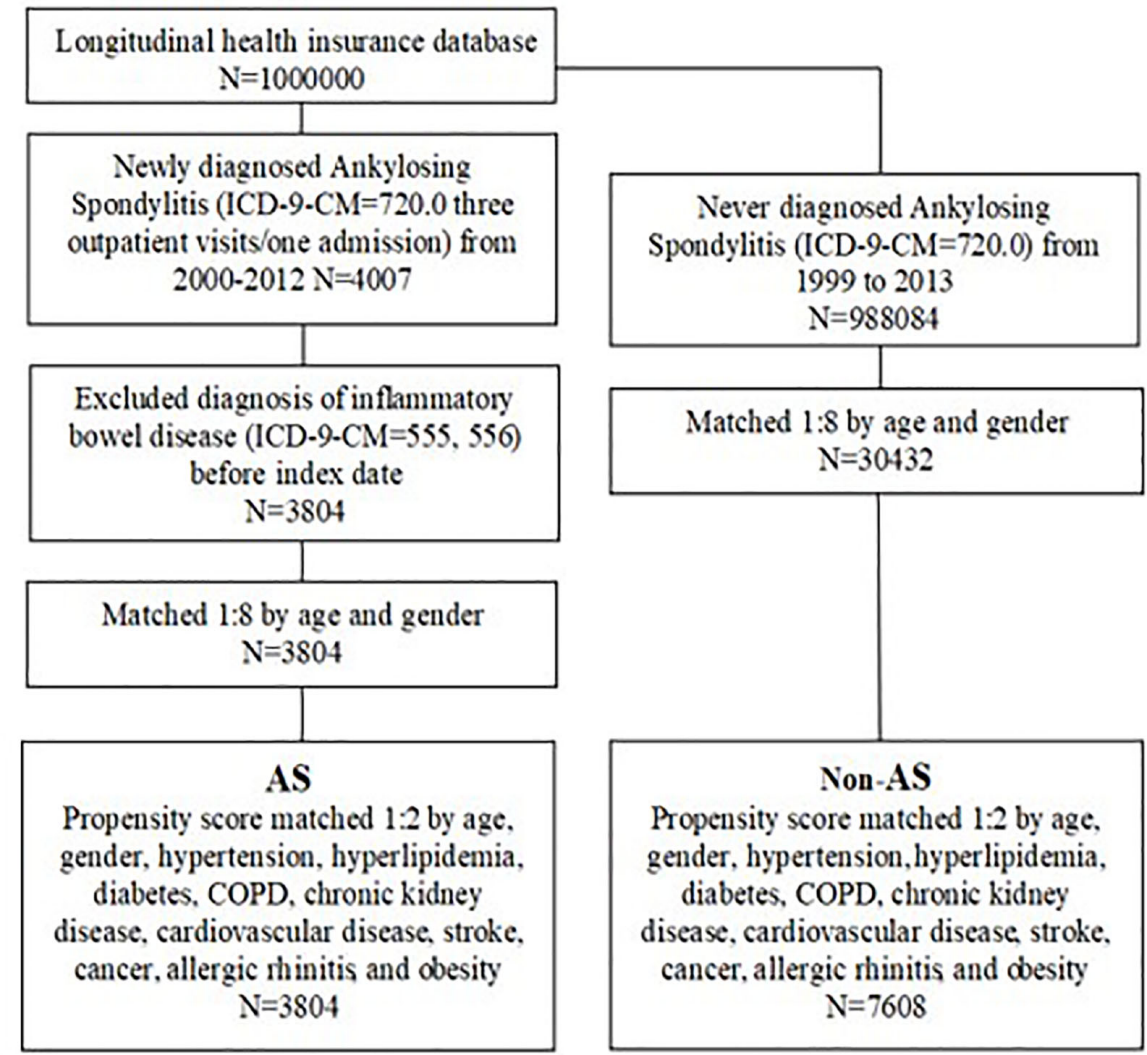
Flowchart of the sample selection.

To reduce selection bias, the AS group was 1:2 matched with the non-AS group by propensity scores based on covariates and baseline characteristics, including age, sex, hypertension (ICD-9-CM 401–405), hyperlipidemia (ICD-9-CM 272.0–272.4), diabetes (ICD-9-CM 250), chronic obstructive pulmonary disease (COPD; ICD-9-CM 490–496), chronic kidney disease (ICD-9-CM 585), cardiovascular disease (ICD-9-CM 410–414), stroke (ICD-9-CM 430–438), cancer (ICD-9-CM 140–208), allergic rhinitis (ICD-9-CM 477), and obesity (ICD-9-CM 278). These comorbidities were defined within 1 year before the index date based on at least three outpatient visits or one hospitalization and were considered covariates in the multivariate analysis. The AS group comprised 3,804 patients and the non-AS group comprised 7,608 patients. **Figure 1** displays the flowchart of the sample selection.

### Statistical Analysis

The chi-square or Fisher exact test were performed to compare categorical variables of the demographic data of AS and non-AS groups, and Student *t* tests were performed to compare continuous variables. A Kaplan–Meier *analysis was* performed to assess *the cumulative incidence of* IBD *and a log-rank test was used to test the significance. A* Cox proportional hazard model was used to estimate the hazard ratio (HR) for IBD between the AS and non-AS groups. Subgroup analyses were also performed by age and sex. The statistical software used was *SPSS (version 18.0; SPSS Inc., Chicago, IL, USA).* Statistical significance was defined as a p of <0.05.

## Results

### Study Population

We identified 4,007 patients with AS with at least three outpatient visits or one hospital admission for AS. We further excluded patients diagnosed as having IBD before to the index date. A total of 11,412 patients were recruited from the claims database, with 3,804 in the AS group and 7,608 in the non-AS group (**Figure 1**).

### Baseline Characteristics of Patients With AS

Among the patients with AS, 38.4% were women and 61.6% were men. Before propensity score matching, the AS group were more likely to have comorbid hypertension (p < 0.001), hyperlipidemia (p = 0.001), diabetes (p = 0.025), COPD (p < 0.001), cardiovascular disease (p < 0.001), stroke (p = 0.023), and allergic rhinitis (p < 0.001) than the non-AS group. By contrast, after propensity score matching, there were no differences in baseline characteristics between the AS and non-AS group, including age, sex, comorbidity hypertension, hyperlipidemia, diabetes, COPD, Chronic kidney disease, cardiovascular disease, stroke, cancer, allergic rhinitis, and obesity (**Table 1**).

**Table 1:** Demographic characteristics of Ankylosing Spondylitis and Non-Ankylosing Spondylitis.

	Before propensity score matched					After propensity score matched				
	Ankylosing spondylitis(N = 3,804)		Non-ankylosing spondylitis(N = 30,432)		p-value	Ankylosing spondylitis(N = 3,804)		Non-ankylosing spondylitis(N = 7,608)		p-value
	n	%	n	%		n	%	n	%	
Age					>0.999					0.997
<40	1,746	45.9	13,968	45.9		1,746	45.9	3,489	45.9	
40–64	1,478	38.9	11,824	38.9		1,478	38.9	2,955	38.8	
≥65	580	15.2	4,640	15.2		580	15.2	1,164	15.3	
Mean ± SD	44 ± 17.7		44 ± 17.7		>0.999	44 ± 17.7		44.2 ± 17.7		0.577
Gender					>0.999					0.663
Female	1,462	38.4	11,696	38.4		1,462	38.4	2,892	38.0	
Male	2,342	61.6	18,736	61.6		2,342	61.6	4,716	62.0	
Hypertension	599	15.7	3,673	12.1	<0.001	599	15.7	1,212	15.9	0.800
Hyperlipidemia	197	5.2	1,228	4.0	0.001	197	5.2	398	5.2	0.905
Diabetes	253	6.7	1,749	5.7	0.025	253	6.7	493	6.5	0.728
COPD	176	4.6	901	3.0	<0.001	176	4.6	323	4.2	0.348
Chronic kidney disease	23	0.6	176	0.6	0.841	23	0.6	43	0.6	0.793
Cardiovascular disease	205	5.4	1,012	3.3	<0.001	205	5.4	403	5.3	0.837
Stroke	112	2.9	714	2.3	0.023	112	2.9	234	3.1	0.699
Cancer	65	1.7	463	1.5	0.377	65	1.7	123	1.6	0.716
Allergic rhinitis	211	5.5	1,004	3.3	<0.001	211	5.5	422	5.5	1.000
Obesity	5	0.1	40	0.1	>0.999	5	0.1	7	0.1	0.549†

^†^Fisher’s exact test.

### HR for IBD

Over the 13 years of follow-up period, 61 patients without and 42 patients with AS were diagnosed as having IBD. This incidence density was 1.0 and 1.4 per 1,000 person-year in patients without and with AS, respectively (**Table 2**). The Cox proportional hazard regression indicated that patients aged ≥65 years [adjusted HR (aHR): 2.48, 95% confidence interval (CI): 1.38–4.47] and with comorbidity of cancer (aHR: 3.51, 95% CI: 1.40–8.80) had a higher HR for IBD. However, no difference was identified in the HR for IBD between the non-AS and AS groups (aHR: 1.37, 95% CI: 0.92–2.02).

**Table 2:** Cox proportional hazard model.

	No. of IBD	Observedperson-years	ID	Crude HR	95% C.I.	Adjusted HR†	95% C.I.
Ankylosing spondylitis							
No	61	60,058	1.0	1		1	
Yes	42	30,188	1.4	1.37	0.93–2.03	1.37	0.92–2.02
Age							
<40	39	43,343	0.9	1		1	
40–64	39	35,371	1.1	1.22	0.78–1.89	1.26	0.79–2.00
≥65	25	11,533	2.2	**2.32**	**1.41**–**3.84**	**2.48**	**1.38**–**4.47**
Gender							
Female	39	33,920	1.1	1		1	
Male	64	56,327	1.1	1.00	0.67–1.48	1.11	0.73–1.66
Hypertension	18	12,359	1.5	1.29	0.78–2.15	0.94	0.52–1.73
Hyperlipidemia	6	3,939	1.5	1.28	0.56–2.92	1.22	0.50–2.96
Diabetes	6	4,796	1.3	1.05	0.46–2.40	0.75	0.31–1.81
COPD	7	3,427	2.0	1.80	0.84–3.88	1.42	0.63–3.20
Cardiovascular disease	5	4,292	1.2	0.99	0.40–2.44	0.65	0.25–1.70
Stroke	2	2,296	0.9	0.74	0.18–3.01	0.50	0.12–2.12
Cancer	5	968	5.2	4.46	1.81–10.97	3.51	1.40–8.80
Allergic rhinitis	6	4,539	1.3	1.13	0.50–2.58	1.05	0.45–2.43

IBD, Inflammatory bowel disease; COPD, chronic obstructive pulmonary disease; ID, Incidence density (per 1,000 person-years).

^†^Adjusted for age, gender, hypertension, hyperlipidemia, diabetes, COPD, cardiovascular disease, stroke, cancer, and allergic rhinitis.

Bold value means these datas have significant difference.

### Time-to-Event Analysis and Subgroup Analysis

**Figure 2:**
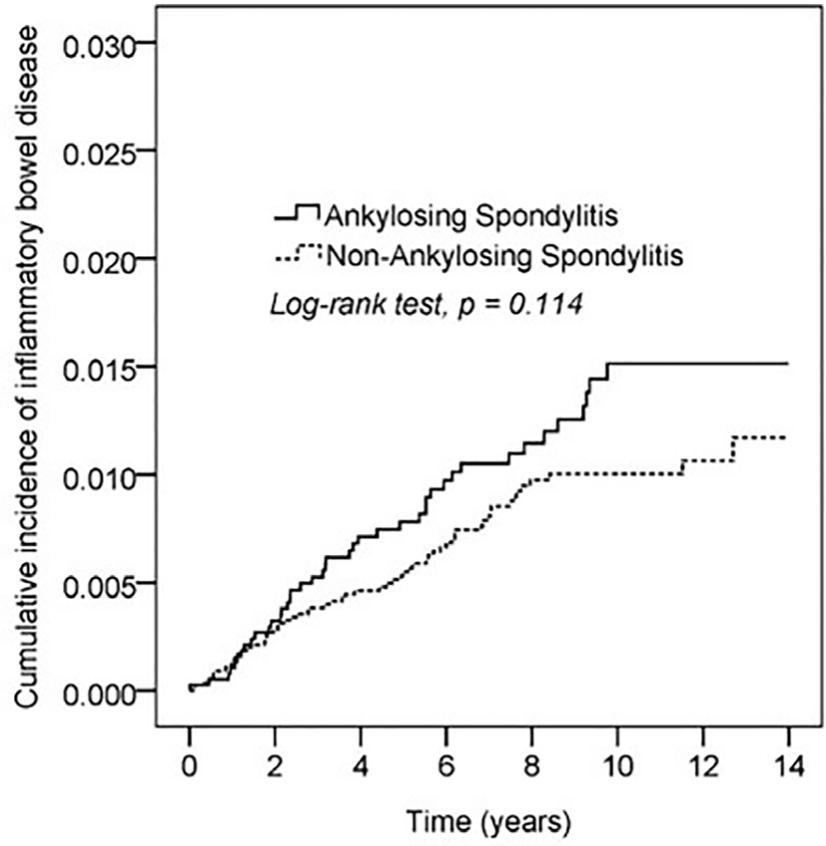
Kaplan-Meier curve of cumulative incidence proportion of IBD in the AS and the comparison cohort population.

**Figure 3:**
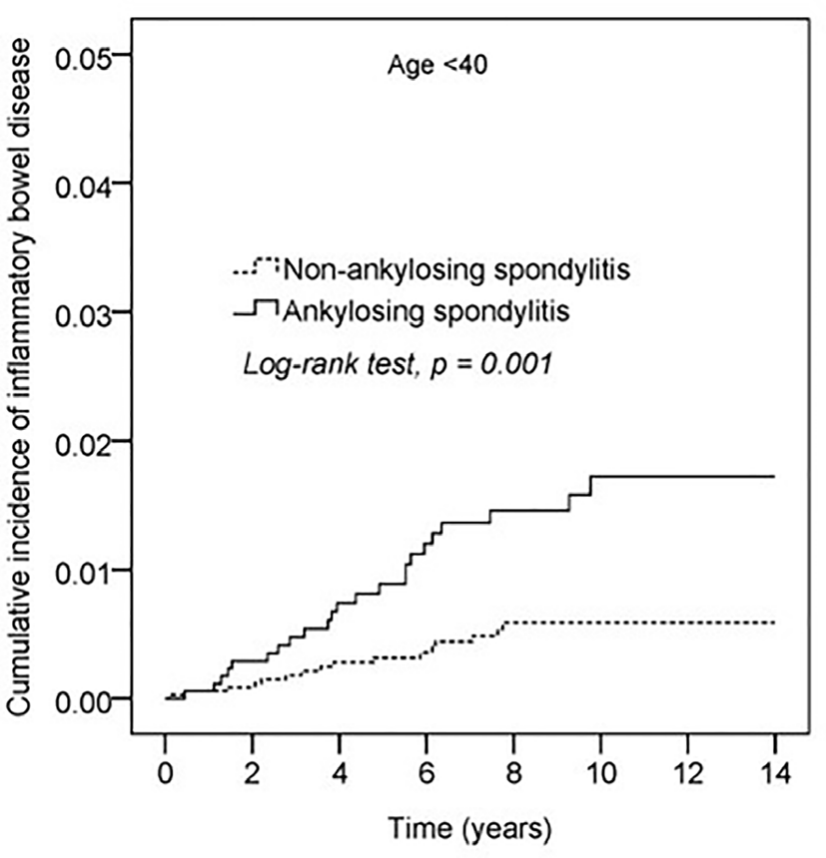
Kaplan-Meier curve of cumulative incidence proportion of IBD in the AS and the non-AS group whose age <40.

Kaplan–Meier curves of the cumulative incidence proportion of IBD are displayed in **Figure 2**; no difference was determined in incidence risk between the AS and non-AS group (log-rank, p = 0.114). The subgroup analyses revealed that patients with AS aged <40 years had a higher HR (2.85, 95% CI: 1.51–5.40) than patients without AS (**Table 3**). Kaplan–Meier curves of cumulative incidence proportion of IBD (age < 40) are displayed in **Figure 3** (p = 0.001).

**Table 3:** Subgroup analysis of Cox proportional hazard model.

	Ankylosing Spondylitis		Non-Ankylosing Spondylitis		HR	95% CI
	N	No. of IBD	N	No. of IBD		
Age						
<40	1,746	23	3,489	16	2.85	1.51–5.40
40–64	1,478	13	2,955	26	1.01	0.52–1.96
≥65	580	6	1,164	19	0.61	0.24–1.52
Gender						
Female	1,462	16	2,892	23	1.37	0.72–2.59
Male	2,342	26	4,716	38	1.37	0.83–2.26

## Discussion

In this population-based cohort study, we determined that patients aged ≥65 years (aHR: 2.48, 95% CI: 1.38–4.47) and patients with cancer (aHR: 3.51, 95% CI: 1.40–8.80) had a higher HR for IBD among the population as a whole. Patients with AS had a higher risk of IBD than non-AS in the subgroup aged <40 years (HR: 2.85, 95% CI: 1.51–5.40, p = 0.001).

A study reported that patients with AS had a higher risk than the general population of developing hypertension, hyperlipidemia, diabetes, cancer, and other cardiovascular, pulmonary, neurological, and renal complications.(6, 7) Concurrently, our findings indicated that patients with AS in Taiwan were more likely to have comorbid hypertension, hyperlipidemia, diabetes, COPD, cardiovascular disease, stroke, and allergic rhinitis at baseline (**Table 1**). Our results showed that, patients with cancer had a higher risk of IBD. It was reported that, the underlying inflammation generated from IBD can promote initate and promote cancer development. Immune cells infiltrate in colon lead to reactive oxygen species and reactive nitrogen species release which cause damage of DNA then initiate cancer. Moreover, inflammatory celss produce growth factors that drive tumor progression.(8, 9)

The highest prevalence of IBD has been reported in Europe (UC 5.1 per 1,000 persons in the southeast of Norway; CD 3.2 per 1,000 persons in Hesse, Germany) and North America (UC 2.8 per 1000 persons in Olmsted County, USA; CD 3.2 per 1,000 persons in Nova Scotia, Canada).(10) We identified a lower incidence rate of IBD in Taiwan; the incidence rate of IBD was 1.0 per 1,000 person-years in patients without AS and 1.4 per 1,000 person-years in Taiwan patients with AS. This may be because of ethnic and environmental differences.

CD and UC reportedly have a bimodal age distribution, with a first peak at approximately 25 years of age and a second peak after 60 years of age, according to population-based studies and hospitalization statistics.(11–15) Our findings were similar. The Cox proportional hazard regression indicated that patients aged ≥65 years had a higher HR for IBD (aHR: 3.14, 95% CI: 1.04–9.44, p < 0.05; **Table 2**). The Cox proportional hazard analysis also indicated that the HR for IBD was higher in patients with comorbid cancer (p < 0.05), with an adjusted HR of 3.51 (95% CI: 1.40–8.77; **Table 2**). This finding is consistent with reports that malignancies such as colorectal cancer, liver cancer, lymphoma, lung cancer, and biliary tract tumor were significantly related to IBD mortality.(16–19)

Stolwijk et al. determined that the incidence and prevalence of IBD were higher in patients with AS than in the general population.(3, 20, 21) Approximately 10% of patients with AS have clinical IBD and up to 70% have subclinical bowel inﬂammation, demonstrated histologically.(22) Our results did not indicate a difference in HRs between AS and non-AS groups. However, the subgroup analysis of the Cox proportional hazard model revealed that patients with AS had a higher risk of IBD than the non-AS group in the subgroup aged <40 years (HR: 2.85, 95% CI: 1.51–5.40; **Table 3**). Kaplan–Meier curves of the cumulative incidence proportion of IBD indicated that patients with AS aged <40 years had a higher risk of IBD than the non-AS group (p = 0.001), as illustrated in **Figure 3**.

In this population-based retrospective cohort study, data were obtained from the NHIRD, which is a peer-reviewed long-term population-based database that covers approximately 99% of the 23 million inhabitants of Taiwan. This is the major strength of our study. However, there are some limitations. First, we used ICD-9-CM diagnostic codes, instead of medical records to confirm diagnoses of IBD and AS. This may have led to the inclusion of misdiagnosed or over-diagnosed cases. However, ICD coding is a catastrophic illness diagnosis and is reviewed by expert clinicians according to Taiwan insurance reimbursement policy, which improves the accuracy. Second, some other confounding factors, such as disease activity, smoking habits, alcohol consumption, and exercise habits, were not collected in this study. To minimize this bias, we stratified lifestyle-related diseases, such as COPD, diabetes, hyperlipidemia as a proxy and propensity score-matched participants to improve baseline comparability. Third, NSAIDs may affect the entire gastrointestinal system, and biologics such as secukinumab, golimumab, and etanercept may increase IBD risk.(23–25) However, we did not collect the data on drug prescriptions. These confounding factors will be included in the study design of our future works.

In conclusion, this large population-based cohort study demonstrated that patients with AS aged <40 years had a higher IBD risk than patients without AS in Taiwan. Physicians managing patients with AS should consider this association when monitoring patients and prescribing medication.

## Data Availability Statement

The raw data supporting the conclusions of this article will be made available by the authors, without undue reservation.

## Ethics Statement

This observational human study protocol and informed consent waiver were reviewed and approved by Chung Shan mMedical University Hospital Institutional Review Board, No. CS15134.

## Author Contributions

SW, JC-C, and ZZ wrote the manuscript. Y-HW analyzed the data. All authors contributed to the article and approved the submitted version.

## Conflict of Interest

The authors declare that the research was conducted in the absence of any commercial or financial relationships that could be construed as a potential conflict of interest.
